# Immunomodulatory roles of quercetin in diabetic nephropathy: targeting inflammation, oxidative stress, and ferroptosis

**DOI:** 10.3389/fphar.2025.1687677

**Published:** 2025-11-13

**Authors:** YaFeng Zheng, XiaoNa Ye, XingJun Han, Wei Geng, Longsheng Zhao, Dandan Meng

**Affiliations:** 1 Department of Preventive Treatment of Disease, The Second Affiliated Hospital of Shandong University of Traditional Chinese Medicine, Jinan, Shandong, China; 2 Department of Traditional Chinese Medicine, Shandong Provincial Maternal and Child Health Care Hospital Affiliated to Qingdao University, Jinan, Shandong, China; 3 School of Continuing Education, Shandong University of Traditional Chinese Medicine, Jinan, Shandong, China; 4 College of Traditional Chinese Medicine, Shandong University of Traditional Chinese Medicine, Jinan, Shandong, China

**Keywords:** quercetin, diabetic nephropathy, natural products, multi-target therapy, drug delivery

## Abstract

Diabetic nephropathy (DN) is one of the most common and disabling chronic complications of diabetes, with a complex pathogenesis involving genetic susceptibility, inflammatory responses, oxidative stress, and other pathological processes. Current therapeutic approaches can partially control hyperglycemia and slow the decline of renal function, but remain insufficient to reverse established structural damage to the kidneys. This underscores the urgent need for novel, safe, and multi-targeted intervention strategies. In recent years, natural bioactive compounds have attracted considerable attention for their potential in preventing and treating chronic diseases. Quercetin, a natural flavonoid widely distributed in plants, exhibits multiple biological activities—including anti-inflammatory, antioxidant, anti-fibrotic, and cell death–modulating effects—and has shown significant promise in DN therapy. This review provides a comprehensive overview of the major pathogenic mechanisms of DN and recent advances in understanding the regulatory effects of quercetin on key pathological processes. We highlight its potential mechanisms of action, including suppression of inflammation and oxidative stress, inhibition of TGF-β1–mediated renal fibrosis, protection of podocyte function, and induction of ferroptosis, and discuss the possible synergistic interactions among these effects in modulating the DN disease network. In addition, we evaluate the current status and limitations of preclinical research on quercetin, and explore feasible strategies—such as nanoparticle-based delivery systems and structural modification—to enhance its bioavailability and tissue targeting. Finally, we propose future research directions for quercetin-based interventions in DN, aiming to provide a theoretical foundation and novel insights for its clinical translation.

## 1 Introduction

Diabetic nephropathy (DN) is a common chronic microvascular complication of diabetes, characterized by pathological features such as glomerulosclerosis, tubulointerstitial fibrosis, and progressive renal insufficiency (Yang and Liu, 2022). The development of DN is primarily associated with structural and functional damage to the kidneys caused by prolonged hyperglycemia, with persistent proteinuria and a gradual decline in renal function being the main clinical manifestations (Samsu, 2021; Ricciardi and Gnudi, 2021). In addition, alterations in renal hemodynamics exacerbate swelling and sclerosis of the glomeruli and renal tubules, further impairing filtration capacity (Tanabe et al., 2020).

Epidemiological studies have demonstrated that DN is a leading cause of chronic kidney failure and end-stage renal disease (ESRD) worldwide (Zou et al., 2022), and is also an important contributor to increased all-cause mortality (Sabanayagam et al., 2019). Compared with other immune-mediated kidney diseases, DN is associated with a higher mortality rate. In recent years, the global prevalence of diabetes has been steadily rising. By 2021, the number of people with diabetes had reached 537 million worldwide, and it is projected to increase to 783 million by 2045, with an alarming trend toward younger onset (Sun et al., 2022; Yu et al., 2024). This growing prevalence not only poses a serious threat to public health but also imposes a substantial socioeconomic and healthcare burden.

The pathogenesis of DN is complex and may be associated with multiple factors, including disturbances in glucose metabolism, oxidative stress, inflammation, and autophagy (Raina et al., 2021; Naaman and Bakris, 2023). Current conventional therapeutic approaches primarily focus on symptomatic management, such as blood pressure control, reduction of proteinuria, and glycemic regulation, often combined with novel hypoglycemic agents. However, these strategies have limited efficacy in preventing disease progression to ESRD, and some pharmacological agents are associated with undesirable adverse effects. Consequently, elucidating the molecular mechanisms underlying DN and identifying safe and effective anti-DN agents remain of critical importance (Kopel et al., 2019). Complementary and alternative medicine (CAM), owing to its abundance of natural bioactive constituents, has emerged as a potential therapeutic strategy. In recent years, natural products have demonstrated unique advantages in DN management, including the ability to slow disease progression, alleviate clinical symptoms, and minimize adverse effects (Yu et al., 2021; Xie et al., 2021).

Quercetin, a naturally occurring flavonoid, is derived from natural sources such as *Ginkgo biloba* and rutin, with the molecular formula C_15_H_10_O_7_. It is also known as 3,3′,4′,5,7-pentahydroxyflavone and is one of the most abundant natural compounds found in plants and fruits. Quercetin exhibits a broad spectrum of pharmacological activities, including antioxidant, anti-inflammatory, hypoglycemic, hypolipidemic, anti–Alzheimer’s disease, anticancer, and antimicrobial effects (Ebrahimpour et al., 2020; Khursheed et al., 2020; Patel et al., 2018; Yang D. et al., 2020; Zou et al., 2021), suggesting its wide therapeutic potential across multiple systems. Emerging evidence indicates that quercetin exerts renoprotective effects in DN through diverse mechanisms, such as attenuation of oxidative stress and inflammation, inhibition of fibrosis, regulation of apoptosis and autophagy, modulation of glucose and lipid metabolism, and induction of ferroptosis (Liu et al., 2024; Jiang et al., 2019; Abdou and Abd elkader, 2022; Lu et al., 2015). Therefore, this review aims to provide a systematic summary of the current research progress on quercetin in the treatment of DN. We first outline the pathogenic mechanisms of DN, followed by an in-depth discussion of the potential molecular pathways through which quercetin exerts its effects, with particular emphasis on key signaling pathways such as Nrf2, NF-κB, TGF-β1/Smad, AMPK/mTOR, and SIRT1. Experimental evidence from both *in vitro* and *in vivo* studies will be reviewed to support these mechanisms. Furthermore, we discuss the existing limitations in current research and propose future directions to improve quercetin’s bioavailability and promote its clinical translation. By elucidating the mechanistic basis of quercetin’s therapeutic effects in DN, this review seeks to provide a scientific foundation for its development as an adjunct or alternative therapeutic strategy for DN management.

## 2 Pathogenesis of diabetic nephropathy

**FIGURE 1:**
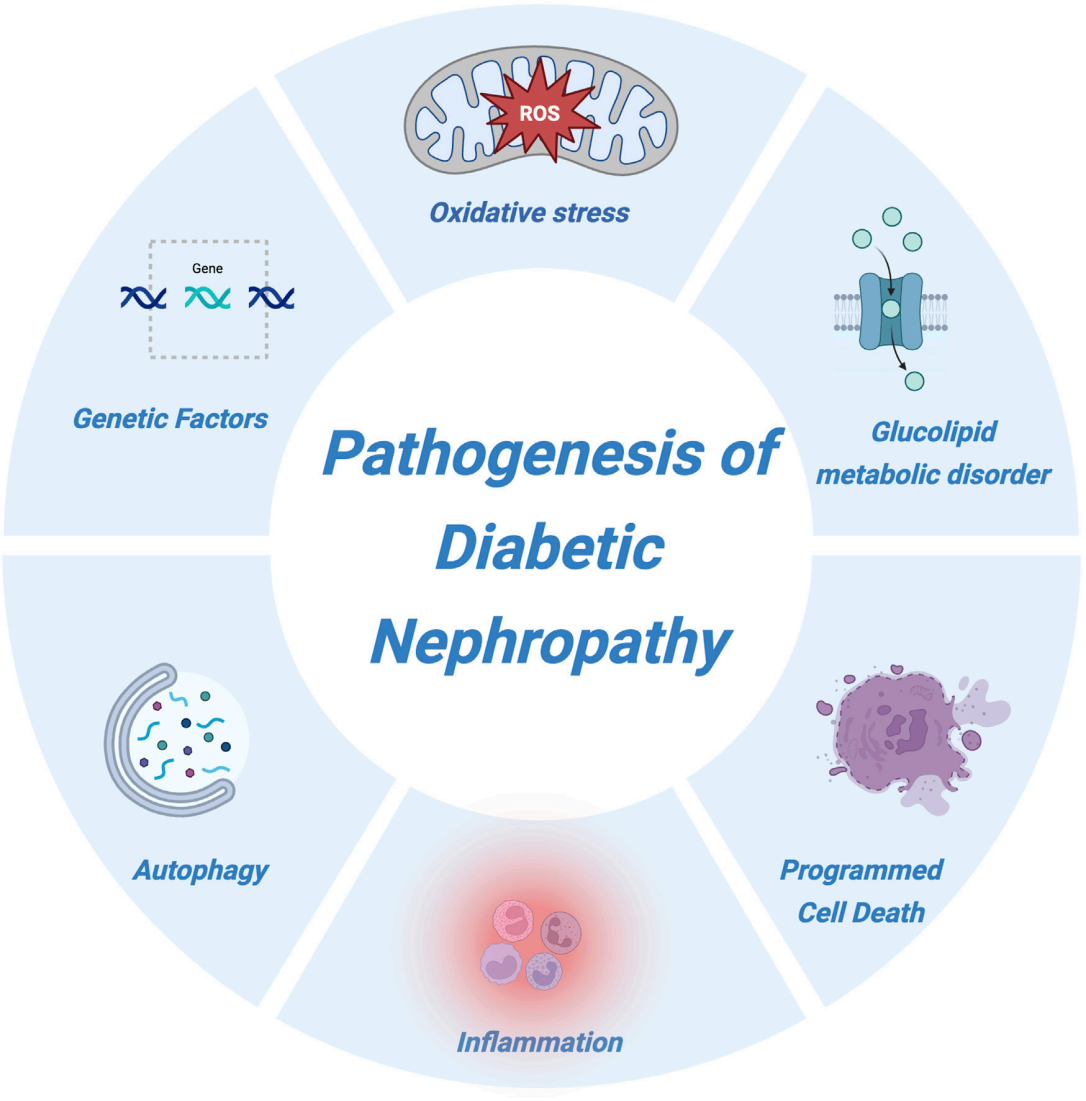
Pathogenesis of diabetic nephropathy. This schematic illustration summarizes the major pathogenic mechanisms involved in the development and progression of diabetic nephropathy. Key contributors include genetic susceptibility, oxidative stress, glucolipid metabolic disorders, inflammation, autophagy dysfunction, and various forms of programmed cell death. These interconnected pathways collectively drive renal injury and accelerate disease progression.

The pathogenesis of DN is highly complex and remains incompletely understood. Current evidence suggests that DN is the result of multiple interacting factors and involves intricate and multifaceted molecular mechanisms. These mechanisms engage numerous signaling pathways, mediators, and interrelated metabolic and hemodynamic variables (Pérez-Morales et al., 2019). Existing studies indicate that the development and progression of DN are closely associated with genetic predisposition, disturbances in glucose and lipid metabolism, hemodynamic abnormalities, oxidative stress, inflammation, and dysregulation of autophagy (Raina et al., 2021). The cumulative effects of these factors over time lead to progressive structural and functional damage to the kidneys. A thorough understanding of the pathogenic mechanisms of DN can help identify key etiological factors, thereby facilitating targeted preventive measures, reducing disease incidence, and ultimately lowering DN-related morbidity and mortality. The pathogenic mechanisms of DN are illustrated in Figure 1.

### 2.1 Genetic factors

Although hyperglycemia is the primary trigger for the onset of DN, not all patients with diabetes progress to this complication. Epidemiological data indicate that approximately 30%–40% of individuals with diabetes will eventually develop DN (Sun et al., 2022), suggesting that genetic factors play a pivotal role in its pathogenesis. The incidence of DN varies significantly among different ethnic groups and families, further supporting the concept of genetic susceptibility (Conserva et al., 2016).

Multiple genes have been implicated in the development of DN, including CARS, PIK3C2G, INPPL1, TBC1D4, N*RXN3*, and *NLRP3*, which have been identified as DN-associated genes in various studies (Pezzolesi and Krolewski, 2013; Saeed, 2018). In addition, single nucleotide polymorphism (SNP) rs4880 has been reported to be associated with diabetes and certain diabetic complications, such as cardiovascular disease, nephropathy, and retinopathy (Yahya et al., 2019). With the advancement of epigenetic research, novel regulatory mechanisms have also been linked to DN, including the involvement of non-coding RNAs, post-translational chromatin modifications, and DNA methylation (Mcknight et al., 2015). These findings indicate that genetic and epigenetic factors jointly contribute to individual susceptibility and disease progression in DN.

### 2.2 Dysregulation of glucose and lipid metabolism

Disorders of glucose and lipid metabolism play a crucial role in the pathogenesis of DN, not only exacerbating glomerular hyperfiltration induced by hyperglycemia but also promoting inflammation, oxidative stress, lipotoxicity, and structural as well as functional renal impairment through multiple metabolic stress pathways. Hyperglycemia is the primary pathogenic factor in DN.

Both hyperglycemia and oxidative stress can lead to abnormal accumulation of extracellular matrix (ECM)–related factors in renal tubular cells, resulting in glomerulosclerosis and tubulointerstitial fibrosis. In the early stages, DN lacks specific clinical manifestations; however, as the disease progresses, patients may develop alterations in glomerular filtration rate, hypertension, and proteinuria, ultimately progressing to ESRD (Sun et al., 2021; Li et al., 2018). Previous studies have reported that more than 80% of biopsy-confirmed DN patients exhibit disturbances in glucose and lipid metabolism (Russo et al., 2020). Persistent hyperglycemia may impair vascular endothelial function, leading to abnormal blood supply to target organs. Moreover, chronic hyperglycemia and glucose fluctuations contribute to excessive lipid accumulation, reduced lipid catabolism, and elevated expression of lipid metabolism–related biomarkers, collectively accelerating renal injury.

Lipids play essential roles in intracellular signal transduction, immune regulation, material transport, and the maintenance of cellular metabolism. Dyslipidemia is one of the hallmarks of diabetes, characterized by abnormal plasma lipid profiles and ectopic lipid accumulation in various tissues and organs, including the kidneys (Thongnak et al., 2020). Such metabolic disturbances may lead to impaired cholesterol metabolism, increased lipid uptake or synthesis, altered fatty acid oxidation, lipid droplet accumulation, and imbalances in bioactive sphingolipids—such as ceramide, ceramide-1-phosphate, and sphingosine-1-phosphate. These changes contribute to renal lipid deposition, glomerulosclerosis, and mesangial expansion, thereby exacerbating proteinuria and the progression of tubulointerstitial fibrosis. In addition, direct podocyte injury may occur, leading to irreversible impairment of renal function (Mitrofanova et al., 2021).

Non-esterified fatty acids (NEFAs) serve as a major energy source for proximal tubular epithelial cells. Fatty acid uptake occurs via endocytosis or through transporters such as CD36 and fatty acid–binding proteins. Excess circulating free fatty acids can downregulate insulin transcription, induce β-cell apoptosis, reduce insulin secretion, and ultimately lead to insulin resistance (Noels et al., 2021; Opazo-Ríos et al., 2020). Recent studies have suggested that, beyond traditional lipid parameters, apolipoprotein A1 (ApoA1), apolipoprotein B (ApoB), and lipid ratios are particularly relevant in patients with type 2 diabetes (Gao et al., 2023). In particular, the low-density lipoprotein (LDL)/ApoB ratio shows a strong correlation with DN, estimated glomerular filtration rate (eGFR), and the urine albumin-to-creatinine ratio (UACR), with lower ratios indicating a higher risk of DN. Furthermore, serum lipoprotein(a) [Lp(a)] and high-density lipoprotein (HDL) levels have also been investigated in relation to DN. Patients with DN exhibit significantly elevated serum Lp(a) levels and reduced HDL levels compared with diabetic patients without nephropathy. Elevated Lp(a) has been identified as a risk factor for DN, whereas HDL appears to exert a protective effect (Mitrofanova et al., 2023).

Collectively, these findings indicate that abnormalities in glucose and lipid metabolism can impair endothelial function, disrupt renal blood supply, and lead to excessive lipid deposition, thereby inducing both structural and functional alterations in renal vasculature and accelerating DN progression. Therefore, regulating glucose and lipid metabolism represents an important therapeutic strategy for the clinical management of DN and for delaying disease progression (Koch et al., 2020).

### 2.3 Oxidative stress

The dynamic balance between cellular oxidation and reduction is fundamental for maintaining cell growth, development, and activity, and is essential for overall health. Oxidative stress refers to a state in which, upon exposure to various harmful stimuli, the body excessively generates reactive oxygen species (ROS) and reactive nitrogen species (RNS), while losing the ability to effectively eliminate these free radicals. This imbalance between oxidant production and antioxidant defenses disrupts tissue and cellular homeostasis, leading to structural and functional impairment (Sies, 2015; Chen et al., 2022). Clinically, oxidative stress is recognized as a major contributor to aging and the development of numerous diseases, underscoring the importance of research on oxidative and antioxidant mechanisms (Ramos-Riera et al., 2023). Under physiological conditions, the oxidative and antioxidant systems remain in dynamic equilibrium to meet normal metabolic demands. However, in patients with diabetes, chronic hyperglycemia exerts deleterious effects on mitochondria, leading to mitochondrial injury and excessive production of ROS and RNS. This disruption of redox balance triggers oxidative stress, thereby contributing to the onset and progression of various diabetic complications, including DN (Zhang Z. et al., 2023).

Oxidative stress represents a converging outcome of multiple pathogenic pathways involved in DN. Hyperglycemia-induced overproduction of ROS is considered a central event in the disease’s pathogenesis. The damage mediated by oxidative stress can occur either directly or indirectly. Directly, ROS injure podocytes, mesangial cells, and endothelial cells, leading to proteinuria and tubulointerstitial fibrosis (Duni et al., 2019). The glomerulus is particularly susceptible to oxidative damage. Hyperglycemia contributes to damage of DNA, lipids, and proteins, and the extent of such injury is closely associated with ROS-induced oxidative stress (Luc et al., 2019).

Oxidative stress is also linked to metabolic and hemodynamic alterations in the kidney, both of which exert synergistic pathogenic effects. Chronic hyperglycemia–induced oxidative stress can elevate angiotensin II (Ang II) levels, activate protein kinase C (PKC), and increase transforming growth factor-β (TGF-β) expression, all of which are recognized as potent pro-oxidative stimuli (Winiarska et al., 2021). Moreover, TGF-β has been shown to participate in NADPH oxidase (NOX)-mediated ROS generation (Wang L. et al., 2021). Sustained overexpression and activation of TGF-β driven by elevated ROS promote excessive ECM remodeling in mesangial cells and accelerate tubulointerstitial fibrosis. Typically, hyperglycemia-induced oxidative stress is thought to enhance pro-inflammatory protein levels by promoting macrophage infiltration. Activated macrophages secrete inflammatory cytokines, thereby driving both local and systemic inflammation. Consequently, oxidative stress is closely interconnected with inflammation and renin–angiotensin–aldosterone system (RAAS) activation, forming a vicious cycle that exacerbates renal injury in DN.

### 2.4 Inflammatory response

Inflammation plays a critical role in the pathogenesis of DN (Moreno et al., 2018; Qiu and Tang, 2016). Rather than being a mere secondary consequence of tissue injury, inflammation represents a key pathophysiological driver of disease onset and progression (Jung and Moon, 2021). Recent studies have highlighted the pivotal role of inflammatory processes in initiating and accelerating DN. Evidence supports the involvement of interleukin (IL)-1, IL-6, and IL-18 in the development and progression of DN (Pichler et al., 2017). Leukocytes, monocytes, and macrophages actively participate in the pathogenic processes of DN, and elevated levels of inflammatory biomarkers are associated with an increased risk of disease. Furthermore, the degree of inflammatory cell infiltration within the kidney strongly correlates with DN severity. In animal models of DN, inhibition of inflammatory cell recruitment to the kidney has been shown to exert renoprotective effects. These findings suggest that inflammation is not only a marker but also a major causal factor in the initiation and progression of DN.

Pro-inflammatory and pro-fibrotic cytokines synthesized and secreted by these infiltrating cells within the local microenvironment can directly disrupt renal architecture, subsequently triggering the epithelial–mesenchymal transition (EMT) process (Liu, 2011), which leads to ECM accumulation. In addition to producing pro-inflammatory cytokines, renal cells in both diabetic animal models and patients exhibit upregulated expression of chemokines and adhesion molecules. These molecules act as key mediators of renal injury by attracting circulating leukocytes and facilitating their transmigration into renal tissue. The infiltrating immune cells serve as an additional source of cytokines and other mediators, thereby contributing to the progression of renal injury and sustaining the inflammatory response. Under hyperglycemic conditions, monocyte chemoattractant protein-1 (MCP-1) secretion is markedly increased in renal tissue, promoting the recruitment of various inflammatory cells. These recruited cells, in turn, secrete large amounts of pro-inflammatory cytokines such as IL-1 and IL-18, creating a vicious cycle of inflammatory injury. Moreover, these inflammatory cells and cytokines can increase urinary albumin excretion and impair renal function (Pérez-Morales et al., 2019).

Taken together, these findings demonstrate that inflammation actively drives the onset and progression of DN, highlighting the damaging effects of hyperglycemia-induced inflammatory responses on the kidney.

### 2.5 Autophagy

Under physiological conditions, autophagy is a protective cellular process that maintains intracellular homeostasis and regulates cell death, playing a critical role in sustaining organismal stability. Both excessive activation and inhibition of autophagy can be detrimental, potentially leading to tissue damage and disease onset (Yu et al., 2023). Studies have shown that in DN model mice, the autophagic activity of intrinsic renal cells—such as podocytes and mesangial cells—is significantly reduced (Kitada et al., 2017; Liu et al., 2018). This decline impairs the timely clearance of damaged proteins and organelles, resulting in renal cell injury, thickening of the glomerular basement membrane (GBM), excessive ECM production, and ultimately promotion of renal fibrosis (Xu et al., 2020). These findings suggest that maintaining normal autophagic activity may represent a promising therapeutic target for DN.

Experimental evidence indicates that modulation of autophagy through upregulation of AMP-activated protein kinase (AMPK) or downregulation of mammalian target of rapamycin (mTOR) can restore autophagic function in DN model mice, increase autophagosome formation, and thereby alleviate renal injury (Sheng et al., 2022; Liu H. et al., 2021). Furthermore, other studies have demonstrated that certain therapeutic agents for DN exert their hypoglycemic effects via regulation of the phosphatidylinositol 3-kinase (PI3K)/protein kinase B (Akt) signaling pathway (Wang et al., 2025; Wu et al., 2025).

Recent studies have revealed that certain non-coding RNAs (ncRNAs) can also influence the development and progression of DN by modulating autophagic activity. Long non-coding RNAs (lncRNAs) constitute the majority of ncRNAs in the genome. For example, lncRNA H19 is highly expressed under hyperglycemic conditions and can suppress podocyte autophagy by downregulating DIRAS3 and subsequently activating the mTOR pathway (Wu and Huang, 2023). Chen et al. (2019) reported that inhibition of the PI3K/Akt/mTOR axis, accompanied by downregulation of miR-142-5p, could activate autophagy and mitigate renal injury.

Taken together, these findings suggest that dysregulated autophagy may play an important role in the onset and progression of DN. Designing precise intervention strategies targeting autophagy regulatory networks—such as inhibiting excessive autophagy in type 1 diabetes mellitus (T1DM) or enhancing lysosomal function in type 2 diabetes mellitus (T2DM)—may provide novel therapeutic approaches to delay DN progression.

## 3 Mechanisms of quercetin in the treatment of DN

### 3.1 Antioxidative stress

Oxidative stress is one of the key mechanisms driving DN progression. It results from excessive accumulation of ROS induced by hyperglycemia through mechanisms such as mitochondrial electron transport chain dysfunction, glucose auto-oxidation, and advanced glycation end-product (AGE) formation. These processes impair the antioxidant defense system, activate inflammatory pathways, and establish a vicious cycle of “oxidative stress–inflammation,” ultimately leading to structural damage in the glomeruli and tubulointerstitium. Prolonged hyperglycemia disrupts the dynamic balance between ROS production and clearance, triggering oxidative stress, which is recognized as an important pathological process in DN (Arellano-Buendía et al., 2014). Under oxidative stress conditions, excessive ROS production is closely linked to DN onset and progression. Elevated ROS can increase glomerular capillary permeability, activate cytokines, impair glomerular filtration function, and induce proteinuria. Therefore, ameliorating oxidative stress represents a promising therapeutic strategy for the prevention and management of DN (Zhou et al., 2019; Du et al., 2018).

Superoxide dismutase (SOD) is a critical ROS scavenger and a key biomarker of oxidative stress, protecting cells from ROS-induced damage. Malondialdehyde (MDA), a major product of lipid peroxidation induced by ROS, can indirectly reflect the level of free radicals and the extent of lipid peroxidation, providing an estimate of the redox balance in plasma (Hofni et al., 2014). In an experimental study using a DN model in C57BL/6J mice, Gomes et al. (2015) demonstrated that low-dose quercetin administration exerted antioxidant, anti-apoptotic, lipid-lowering, and renoprotective effects. These findings suggest that the antioxidative properties of quercetin contribute significantly to its therapeutic potential in DN.

Recent studies have demonstrated that dysregulation of microRNAs is closely associated with renal diseases. It has been reported that overexpression of miR-370 and miR-217 contributes to high glucose (HG)-induced podocyte injury, thereby promoting the development of DN (Xian et al., 2018; Sun et al., 2017). Building on this evidence, Wan et al. (2022) investigated the role of miR-485-5p in DN. Their study revealed that quercetin suppressed HG-induced proliferation, inflammation, and oxidative stress in human mesangial cells (HMCs). Moreover, serum samples from DN patients showed downregulation of miR-485-5p and upregulation of Yes-associated protein 1 (YAP1). Mechanistically, quercetin exerted its protective effects by modulating the miR-485-5p/YAP1 axis to attenuate HG-induced oxidative stress, suggesting a novel therapeutic strategy for DN.

Transforming growth factor-β1 (TGF-β1) upregulation occurs in nearly all kidney diseases and serves as a central mediator of podocyte injury through both Smad-dependent and Smad-independent pathways (Herman-Edelstein et al., 2013). Smad proteins are transcription factors that mediate TGF-β signaling by forming complexes with one another. The generation of ROS has been shown to activate the TGF-β1/Smad signaling pathway and induce podocyte injury (Chang et al., 2016; Yu et al., 2010). Therefore, inhibition of oxidative stress and TGF-β1/Smad pathway–mediated podocyte damage may represent a potential therapeutic target for DN.

In an *in vivo* study, Gao et al. (2018) reported that quercetin ameliorated podocyte injury in DN rats by reducing the expression of nephrin, an effect potentially associated with the suppression of oxidative stress and the TGF-β1/Smad signaling pathway. Specifically, quercetin increased SOD and glutathione (GSH) levels while reducing MDA levels. Moreover, quercetin inhibited TGF-β1–induced phosphorylation of Smad2 and Smad3 in the kidneys of DN rats. Interestingly, quercetin treatment also markedly upregulated Smad7, a negative regulator of TGF-β1 signaling. Collectively, these findings indicate that quercetin protects podocytes in DN by attenuating oxidative stress and inhibiting the TGF-β1/Smad pathway. This provides promising new evidence supporting the potential application of quercetin in maintaining podocyte integrity and preventing proteinuria. The mechanisms by which quercetin alleviates oxidative stress in the treatment of DN.

### 3.2 Anti-inflammatory

Inflammation is one of the central drivers of DN progression. Hyperglycemic stimulation can induce the production of large amounts of pro-inflammatory cytokines in renal tissue, activate immune cells, and ultimately lead to glomerulosclerosis and tubulointerstitial fibrosis. Infiltration of inflammatory cells such as lymphocytes, neutrophils, and macrophages has been shown to contribute to renal injury in DN (Wang et al., 2021b). Quercetin, as a natural flavonoid, has demonstrated clear anti-inflammatory properties in multiple studies (Tang et al., 2019; Li et al., 2020). Clinical and experimental evidence indicates that interleukin-6 (IL-6) and tumor necrosis factor-alpha (TNF-α) are significantly associated with proteinuria in DN patients. Upregulation of TNF-α can enhance local production of ROS, exacerbate oxidative stress–induced renal injury, and trigger the release of other inflammatory cytokines, including IL-6. This cascade promotes inflammatory cell infiltration and endothelial dysfunction (Sathibabu Uddandrao et al., 2019).

Abdou and Abd elkader (2022) evaluated the antidiabetic, antioxidant, and anti-inflammatory effects of quercetin extracted from *Trifolium alexandrinum* extract (TAE) in a streptozotocin (STZ)-induced DN model. Compared with the untreated STZ-diabetic group, quercetin treatment significantly reduced renal levels of TGF-β, TNF-α, and IL-6, improved renal function, and alleviated inflammatory damage. These findings suggest that the renoprotective effects of quercetin may be mediated, at least in part, by its anti-inflammatory properties. Both quercetin and TAE-derived formulations hold promise as potential therapeutic agents for DN with translational clinical value.

Yes-associated protein (YAP), also known as YAP1, is a transcriptional coactivator that functions as a critical molecular switch and downstream effector in the Hippo signaling pathway (Yagi et al., 1999; Ramos and Camargo, 2012). Previous studies have demonstrated that YAP1 is closely associated with the onset and progression of DN (Huang et al., 2018; Ma et al., 2019; Yang T. et al., 2020). In an experimental study, Wan et al. (2022) investigated the effects of quercetin on cell proliferation, inflammatory cytokines (TNF-α, IL-1β, and IL-6), and YAP1-related expression. They found that quercetin suppressed HG-induced proliferation and inflammatory responses in HMCs. Enzyme-linked immunosorbent assay (ELISA) results showed that the protective effects of quercetin on stress-induced inflammatory cytokines (TNF-α, IL-1β, and IL-6) were largely abolished by a miR-485-5p inhibitor. Mechanistically, miR-485-5p directly binds to YAP1 and inhibits its expression. These findings indicate that quercetin exerts anti-inflammatory effects against HG-induced injury by regulating the miR-485-5p/YAP1 axis.

Yin Yang 1 (YY1) is a nuclear transcription factor that plays a pivotal role in regulating cellular metabolism (Zhang et al., 2017). Recent studies have demonstrated that upregulation of YY1 contributes to renal fibrosis in DN (Du et al., 2021; Yang et al., 2023a). Increasing evidence further suggests that YY1 functions as a novel pro-inflammatory mediator involved in various inflammation-related diseases (Mu et al., 2020; Lin et al., 2021). Based on this, Yang et al. (2023b) investigated the critical anti-inflammatory role of quercetin in DN-associated tubulointerstitial inflammation. They found that in diabetic models (db/db mice and HG–treated HK-2 cells), YY1 expression was markedly elevated, which promoted activation of the IL-6/STAT3 inflammatory pathway and exacerbated tubulointerstitial inflammation. Quercetin was shown to bind directly to YY1, reducing its protein expression. Mechanistically, YY1 binds to the promoter region of *IL-6*, directly suppressing its transcription. By directly inhibiting the inflammatory transcription factor YY1, quercetin downregulated the IL-6/STAT3 pathway, thereby significantly alleviating tubulointerstitial inflammation in DN models. These findings reveal a novel mechanism by which quercetin exerts therapeutic effects against DN and provide a mechanistic basis for developing quercetin as a targeted anti-DN agent.

Macrophages are a crucial component of the innate immune system, playing essential roles in immune regulation, inflammatory responses, and tissue repair (Locati et al., 2020; Park et al., 2022). Recent evidence indicates that imbalanced macrophage polarization is increasingly recognized as a major driver of inflammatory responses and renal fibrosis in DN (Landis et al., 2018; Klessens et al., 2017). In the early stages of DN, pro-inflammatory M1 macrophages exacerbate renal tubular epithelial cell injury and initiate renal inflammation through the secretion of pro-inflammatory cytokines. In contrast, anti-inflammatory M2 macrophages promote tissue repair and resolution of inflammation by secreting anti-inflammatory mediators such as arginase-1 (Arg-1) and interleukin-10 (IL-10) (Gu et al., 2024). During DN progression, excessive activation of M1 macrophages sustains chronic renal inflammation and fibrosis (Calle and Hotter, 2020). A recent study (Abudoureyimu et al., 2025) demonstrated that quercetin markedly reduced the expression of NLRC5, NLRP3, TNF-α, IL-6, and IL-1β in HG-stimulated RAW 264.7 macrophages *in vitro*, while increasing the expression of M2-associated markers including CD206, Arg-1, and IL-10. In DN mice, quercetin treatment improved renal histopathological injury and fibrosis, significantly downregulated NLRC5, NLRP3, Col1a1, and α-SMA expression, and decreased renal levels of TNF-α, IL-6, and IL-1β.

**FIGURE 2:**
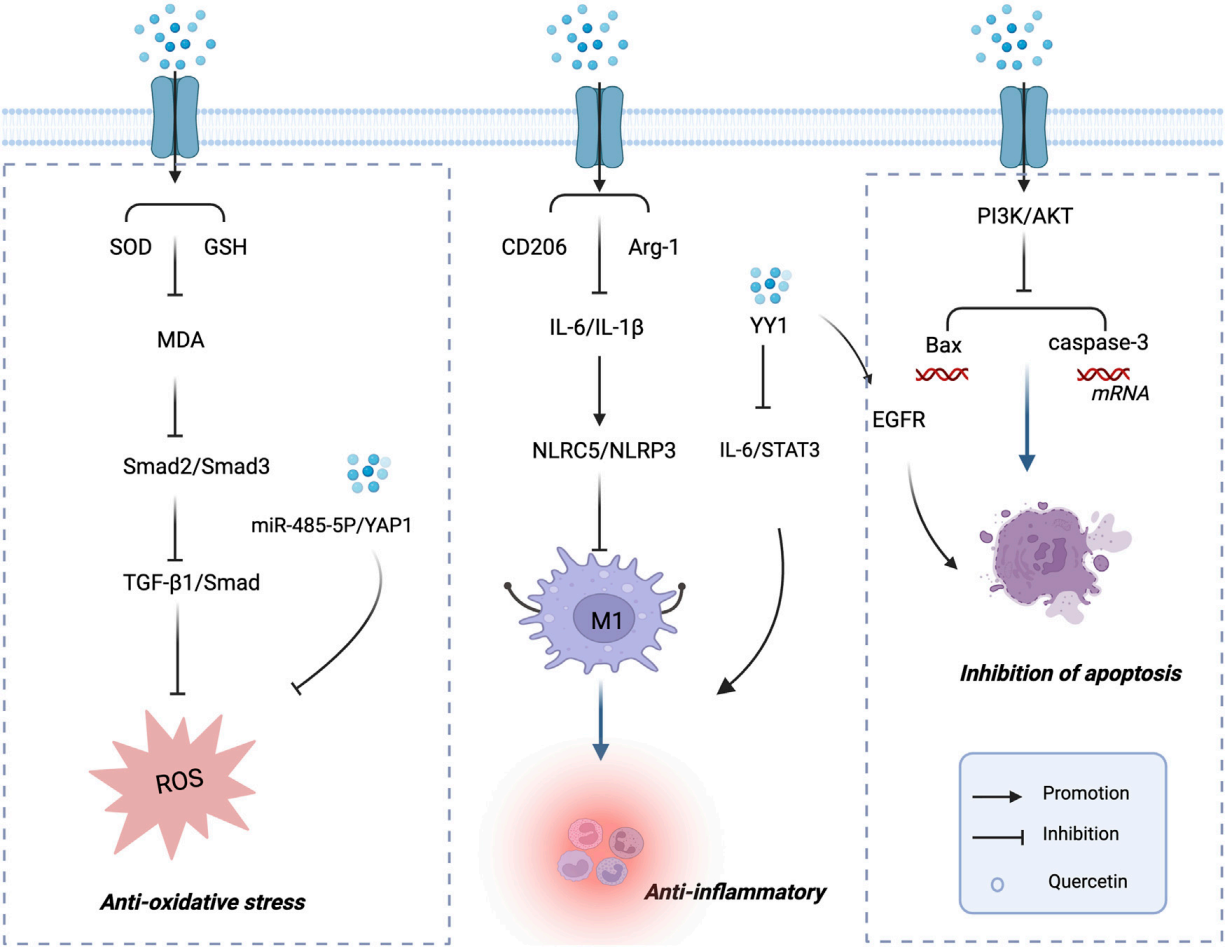
Mechanisms of Quercetin in the Treatment of DN. Mechanisms of quercetin in the treatment of diabetic nephropathy. This schematic illustration depicts the major mechanisms by which quercetin exerts renoprotective effects in diabetic nephropathy. Quercetin alleviates oxidative stress by enhancing SOD and GSH activity and reducing MDA, thereby inhibiting the Smad2/3 and TGF-β1/Smad signaling pathways. It suppresses inflammatory responses by modulating CD206, Arg-1, IL-6, and IL-1β, leading to the regulation of NLRC5/NLRP3 inflammasome activation and IL-6/Stat3 signaling. In addition, quercetin inhibits apoptosis via the PI3K/AKT pathway, downregulating Bax and caspase-3 expression while enhancing EGFR signaling. These multi-targeted actions highlight the therapeutic potential of quercetin in preventing renal injury and slowing the progression of diabetic nephropathy.

These findings suggest that quercetin ameliorates DN by targeting the NLRC5/NLRP3 pathway to suppress M1 macrophage polarization while promoting an anti-inflammatory M2 phenotype. This highlights a novel molecular target for DN treatment and underscores quercetin’s potential as a promising therapeutic candidate for DN. The mechanisms by which quercetin exerts anti-inflammatory effects in the treatment of DN are illustrated in Figure 2.

### 3.3 Inhibition of podocyte apoptosis

Apoptosis plays a crucial role in maintaining cellular homeostasis throughout growth and development, and its dysregulation is a recognized risk factor for multiple diseases (Godse et al., 2017). Numerous studies have demonstrated that suppressing tubular epithelial cell apoptosis can effectively ameliorate DN (Wang et al., 2022; Xing et al., 2021), further supporting the therapeutic relevance of targeting apoptosis in DN prevention and treatment. Liu et al. (2024), through both *in vivo* and *in vitro* experiments, found that in the diabetic mellitus (DM) group, mRNA and protein levels of BAX, as well as cleaved caspase-3 (c-Caspase3), were significantly elevated, with a concomitant increase in the BAX/BCL-2 ratio—indicating enhanced pro-apoptotic signaling. Conversely, quercetin intervention markedly inhibited diabetes-associated renal cell apoptosis, evidenced by reduced *Bax* and *Caspase-3* mRNA levels, decreased BAX and c-Caspase3 protein expression, and a lowered BAX/BCL-2 ratio. Mechanistically, these effects were mediated via activation of the PI3K/AKT pathway, which suppressed tubular epithelial cell apoptosis. Collectively, these findings suggest that quercetin prevents DN progression by attenuating tubular epithelial cell apoptosis through PI3K/AKT signaling modulation, highlighting its potential as a promising candidate for DN prevention.

Podocyte apoptosis is one of the key mechanisms underlying the transition from compensatory hypertrophy to detachment during podocyte injury. This process is primarily characterized by the formation of apoptotic bodies and is accompanied by upregulated expression of pro-apoptotic proteins such as Bax and cleaved caspase-3. Previous studies have shown that pretreatment with *Abelmoschus manihot* total flavonoids (TFA) markedly reduces the number of apoptotic podocytes in DN rats, decreases the expression of pro-apoptotic proteins caspase-3 and caspase-8, thereby attenuating proteinuria and improving renal function (Zhou et al., 2012). These findings suggest that inhibiting podocyte apoptosis is a critical step in alleviating podocyte injury and reducing proteinuria. Liu Y. et al. (2021) investigated the relationship between the epidermal growth factor receptor (EGFR) signaling pathway and the protective effects of quercetin against podocyte apoptosis. They found that in HG-induced podocytes and diabetic mice, the expression of Bax and c-Caspase3 was significantly upregulated, whereas Bcl-2 expression was markedly downregulated. Quercetin treatment reversed these alterations, indicating that quercetin attenuates podocyte apoptosis under diabetic conditions.

Previous research has demonstrated that inhibition of the EGFR pathway can ameliorate renal injury by reducing inflammation, oxidative stress, apoptosis, and fibrosis both *in vivo* and *in vitro* (Skibba et al., 2016). Consistent with these findings, Liu et al. reported that quercetin suppresses Bax and caspase-3 expression via inhibition of EGFR signaling. Collectively, these results highlight quercetin as a natural compound with significant therapeutic potential in DN, exerting its renoprotective effects, at least in part, through suppression of the EGFR-mediated apoptotic pathway.

Network pharmacology, as an auxiliary approach, enables the elucidation of drug mechanisms from a holistic perspective, breaking through the traditional “one drug–one target” paradigm and achieving multi-target synergistic effects (Zhang et al., 2019). Ma et al. (2022) employed network pharmacology, microarray data analysis, and molecular docking to investigate the protective mechanism of quercetin against podocyte injury in order to attenuate DN. Using this integrative strategy, they identified three key targets—TNF, VEGFA, and AKT1—and the advanced glycation end products–receptor for AGE (AGE–RAGE) signaling pathway as the primary mechanism implicated in diabetic complications.

Molecular docking models demonstrated that QUE exhibited strong binding affinities with these key targets. Further experiments revealed that QUE exerts its protective effects on DN-associated podocyte injury through multiple mechanisms: (i) downregulating the pro-inflammatory cytokine TNF, (ii) inhibiting apoptosis via stimulation of AKT1 phosphorylation, and (iii) suppressing AGE-induced oxidative stress by modulating the AGE–RAGE signaling pathway. These findings provide a scientific basis for the development of QUE as a potential natural therapeutic agent for DN. The mechanisms by which quercetin protects podocyte structure and function in diabetic nephropathy are illustrated in Figure 2.

### 3.4 Anti-fibrotic

The late-stage pathological changes of DN are characterized by glomerulosclerosis and tubulointerstitial fibrosis, with the progression of fibrosis being a critical factor leading to irreversible loss of renal function. Current evidence indicates that renal fibrosis is a major driver of DN progression toward ESRD (Zheng et al., 2021). Among the various mechanisms involved, EMT represents a pivotal step in the development and progression of renal fibrosis. EMT refers to the process whereby epithelial cells gradually lose their characteristic epithelial phenotype and acquire features of fibroblasts. This transition is typified by the downregulation of epithelial markers such as E-cadherin, concomitant with the upregulation of EMT-associated markers including collagen type III alpha 1 chain (Col3α1) and vimentin (Zhang HF. et al., 2023; Chen et al., 2024).

Lu et al. (2015) demonstrated, in both *in vitro* and *in vivo* experiments, that HG significantly induces EMT in HK-2 and NRK-52E tubular epithelial cells, accompanied by activation of the mTORC1/p70S6K signaling pathway, including phosphorylation of mTOR and p70S6K. Quercetin treatment reversed these changes by increasing E-cadherin expression while decreasing α-SMA and vimentin levels. Furthermore, quercetin suppressed mTOR and p70S6K phosphorylation, thereby blocking EMT signaling. In STZ-induced diabetic rats, quercetin markedly inhibited mTORC1/p70S6K activation in the renal cortex and alleviated histological signs of fibrosis. Mechanistically, quercetin appears to exert its anti-fibrotic effects by targeting the mTORC1/p70S6K pathway, effectively suppressing EMT in tubular epithelial cells, and preventing the upregulation of EMT transcription factors such as Snail and Twist. This, in turn, significantly mitigates DN-associated tubulointerstitial fibrosis and functional impairment.

In addition, studies have demonstrated that quercetin modulates the TGF-β1/Smad signaling pathway, which is recognized as a central molecular axis in the development and progression of renal fibrosis. In diabetic models, quercetin markedly downregulates the expression of TGF-β1 and the phosphorylation of Smad2/3, while restoring the expression of the inhibitory regulator Smad7 (Gao et al., 2018; Lai et al., 2012). Through these actions, quercetin effectively blocks TGF-β1–mediated ECM synthesis and EMT. Furthermore, quercetin activates the Nrf2/HO-1 antioxidant signaling pathway, thereby reducing ROS-induced TGF-β1 overexpression and indirectly suppressing the activation of fibrogenic signaling (Feng et al., 2023).

Glomerulosclerosis and tubulointerstitial fibrosis are two major pathological hallmarks of DN (Mori et al., 2021; Ruiz-Ortega et al., 2020), both of which are closely associated with renal inflammation. Yang et al. (2023b) reported that the expression of Yin Yang 1 (YY1) was markedly upregulated in HG-treated HK-2 tubular epithelial cells and in the kidneys of *db/db* diabetic mice, accompanied by increased α-SMA expression and activation of the EMT process, thereby aggravating tubulointerstitial fibrosis. Quercetin treatment directly bound to YY1, downregulating its expression and subsequently reducing the levels of fibrosis markers such as α-SMA and vimentin. In addition, quercetin suppressed the upstream activation of YY1 by blocking the mTORC1/p70S6K signaling pathway, thereby attenuating EMT and tubulointerstitial fibrosis.

**FIGURE 3:**
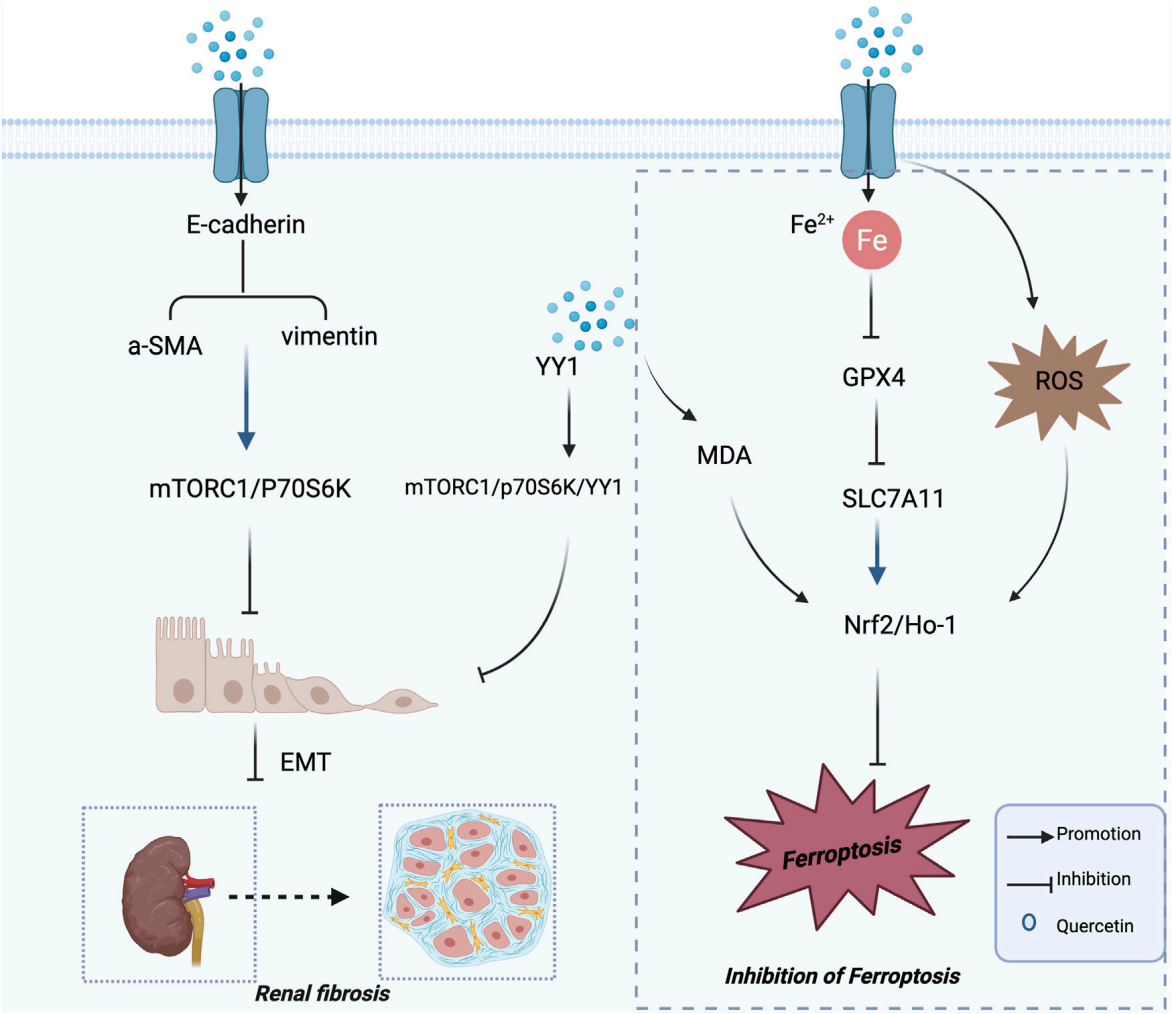
Mechanisms of Quercetin in the Treatment of DN. Mechanisms of quercetin in attenuating renal fibrosis and ferroptosis in diabetic nephropathy. This schematic illustration highlights the dual mechanisms by which quercetin exerts renoprotective effects in diabetic nephropathy. On the left, quercetin suppresses renal fibrosis by downregulating α-SMA and vimentin, inhibiting the mTORC1/p70S6K and YY1 signaling pathways, and preventing EMT. On the right, quercetin attenuates ferroptosis by regulating Fe^2+^ homeostasis, enhancing GPX4 and SLC7A11 expression, and activating the Nrf2/HO-1 signaling pathway, thereby reducing lipid peroxidation and ROS accumulation. Collectively, these actions contribute to the inhibition of renal injury and disease progression in diabetic nephropathy.

In *in vivo* experiments, quercetin administration in diabetic mice markedly increased the expression of E-cadherin and ZO-1, while reducing the expression of α-SMA, vimentin, Snail, and Twist in the renal cortex. Further mechanistic investigations revealed that quercetin effectively inhibited YY1-mediated EMT and ECM protein production by suppressing the mTORC1/p70S6K–YY1 pathway, thus significantly alleviating DN-associated tubulointerstitial fibrosis. This mechanistic evidence provides a clear molecular basis for quercetin’s anti-fibrotic activity and suggests a potential pharmacological strategy for targeting DN. Collectively, these findings offer valuable insights into the therapeutic potential of quercetin in clinical DN management. The mechanisms by which quercetin inhibits renal fibrosis in the treatment of DN are illustrated in Figure 3.

### 3.5 Induction of ferroptosis

Ferroptosis is a recently identified form of regulated cell death characterized by iron-dependent lipid peroxidation, involving a dynamic imbalance among iron metabolism, lipid peroxidation, and antioxidant defense systems (Dixon et al., 2012). An increasing body of evidence suggests that ferroptosis plays a role in a variety of human diseases, including those affecting the kidney (Tonnus et al., 2021; Li et al., 2021). Recent studies have highlighted its contribution to tubular cell injury observed in DN (Kim et al., 2021). Therefore, inhibition of ferroptosis has emerged as a potential therapeutic strategy for the management of DN. Quercetin, a natural flavonoid compound, exerts multiple biological activities, including potent antioxidant effects. Its antioxidative properties are mediated through several mechanisms, such as increasing GSH levels, enhancing antioxidant signaling pathways, and mitigating reactive ROS induced oxidative damage (Feng et al., 2023; Xu et al., 2019). Notably, recent research indicates that quercetin can chelate iron, which may contribute to its renoprotective effects in both acute kidney injury and DN in *db/db* mouse models (Feng et al., 2023; Wang et al., 2021c). These findings suggest that quercetin’s ability to modulate ferroptosis could represent an important mechanism underlying its therapeutic potential in DN.

Transcription factor nuclear factor erythroid 2–related factor 2 (Nrf2) is a master regulator of cellular antioxidant responses and plays a pivotal role in controlling ROS levels and preventing ferroptosis (Dodson et al., 2019). Zhang et al. (2024) investigated the effects of quercetin on HG-induced renal tubular epithelial cell injury and its regulation of Nrf2 in a DN rat model, with a focus on the underlying molecular mechanisms. Their results showed that elevated glucose levels promoted the progression of ferroptosis in the kidneys of DN rats. Furthermore, they observed that Nrf2 expression was reduced in DN renal tissues compared with controls, whereas treatment with quercetin or ferrostatin-1 (Fer-1, a ferroptosis inhibitor) restored Nrf2 expression. These findings indicate that quercetin protects against diabetic renal injury by activating the Nrf2 signaling pathway to inhibit ferroptosis. Overall, this study highlights the therapeutic potential of quercetin in DN and provides novel insights into its renoprotective mechanisms.

Feng et al. (2023) conducted both *in vivo* and *in vitro* experiments and observed increased intracellular iron levels, decreased expression of glutathione peroxidase 4 (GPX4) and solute carrier family 7 member 11 (SLC7A11), as well as elevated MDA and ROS levels in DN models, indicating a significant involvement of ferroptosis in disease progression. Administration of quercetin or a ferroptosis inhibitor significantly reversed these abnormalities. *In vitro*, quercetin upregulated Nrf2 and HO-1, thereby activating the Nrf2/HO-1 pathway (Feng et al., 2023). Activated Nrf2 coordinates antioxidant and iron-handling programs—acting together with HO-1, ferritin (FTH1), and the iron exporter ferroportin (SLC40A1)—to restrict the labile iron pool and suppress lipid peroxidation (Dodson et al., 2019). Notably, Nrf2 directly binds antioxidant response elements (AREs) within the SLC7A11 promoter and, in specific cellular contexts, has also been shown to bind the GPX4 promoter, enhancing its transcriptional activity and thereby maintaining glutathione homeostasis and promoting the clearance of lipid peroxides (Feng et al., 2021; Gu et al., 2023). Consequently, quercetin suppresses tubular epithelial ferroptosis through coordinated activation of the Nrf2–HO-1–GPX4/SLC7A11 axis, elucidating its nephroprotective mechanism in DN and supporting its promise as a ferroptosis-targeting therapeutic candidate (Li et al., 2021). The mechanisms by which quercetin regulates ferroptosis in the context of diabetic nephropathy are illustrated in Figure 3, and the overall mechanisms of quercetin in the treatment of diabetic nephropathy are summarized in Table 1.

**TABLE 1:** Mechanisms of quercetin in the treatment of DN.

Mechanism	Model/Tissue	Concentration	Duration of administration	Target	Ref
Antioxidation	HMCs	10,20, 40 μM	48 h	miR-485-5p/YAP1	Wan et al. (2022)
	db mice	50 and 100 mg kg^-1^	12w	SOD、MDA TGF-β1/Smad	Gao et al. (2018)
Anti-inflammatory	db mice	50 mg/kg	5w	TGF-β、TNF-α、IL-6	Abdou and Abd Elkader (2022)
	HMCs	10、20、40 μM	48 h	TNF-α、IL-1β、IL-6 miR-485-5p/YAP1	Wan et al. (2022)
	db mice	50、100、150 mg/kg	8w	IL-6/STAT3 、YY1	Yang et al. (2023b)
	DN mice	50、100 mg/kg	12w	NLRC5/NLRP3 TNF-α、IL-6、IL-1β	Abudoureyimu et al. (2025)
Anti-apoptosis	T2DM model	150 mg/(kg•bw•d)	4w	PI3K/AKT BAX、c-Caspase3、	Liu et al. (2024)
	db mice	50、100、150kg^−1^	8w	Bax、cleaved caspase-3、EGFR	Liu et al. (2021b)
Anti-fibrosis	db mice	30, 60,90 mg/Kg	14w	mTORC1/p70S6K、E-cadherin、α-SMA	Lu et al. (2015)
	HK-2	10、20、40 μM/L	72 h	α-SMA、vimentin、 mTORC1/p70S6K, mTORC1/p70S6K-YY1	Yang et al. (2023b)
Induction of ferroptosis	HK-2	0, 5, 10, 15, 25, 50 μM	48 h	Nrf2 MDA、GSH	Zhang et al. (2024)
	HK-2	6.25, 12.5, 25, 50, 100 μM	48 h	Nrf2/HO-1,GPX4、SLC7A11、MDA、ROS	Feng et al. (2023)

Although the present review focuses on inflammation, oxidative stress, and ferroptosis, accumulating evidence indicates that dietary phenolics—including quercetin—exert hypoglycemic and lipid-regulating effects that can secondarily mitigate renal lipid deposition in DN (Sarkar et al., 2021; DE Paulo Farias et al., 2021). For completeness, we cite a broader review on functional-food phenolics supporting these metabolic actions (Golovinskaia and Wang, 2023), while a systematic appraisal of metabolic endpoints remains beyond the prespecified scope of this manuscript.

### 3.6 Consistency of Quercetin’s efficacy across different DN models

Given that different DN models capture distinct facets of the human disease, we compared quercetin’s efficacy across STZ-induced rats, db/db mice, and HFD + STZ models to assess cross-model consistency. Overall, quercetin consistently suppressed TGF-β1/Smad signaling, attenuated EMT/ECM accumulation, and activated Nrf2/HO-1, as evidenced in STZ-induced and db/db models (Gao et al., 2018; Lai et al., 2012; Feng et al., 2023). In addition, inhibition of NF-κB–mediated inflammation has been observed in multiple models, suggesting shared upstream regulatory effects (Yang et al., 2023b).

Comparative analyses across models further show convergent renal protection, including improvements in renal histopathology and reductions in serum creatinine and urea nitrogen (Gao et al., 2018; Lai et al., 2012). Nonetheless, certain discrepancies exist: quercetin’s modulation of AMPK or PI3K/Akt appears model-dependent, likely reflecting differences in insulin-resistance severity, metabolic background, and dosing regimens (Yang et al., 2023b). Taken together, the available evidence indicates that quercetin exhibits a stable and reproducible therapeutic effect across diverse DN models, supporting its potential as a broadly applicable candidate for DN intervention.

### 3.7 Dose–response relationship and safety

Across DN models, quercetin tends to show a dose–dependent pattern, whereby higher doses are associated with greater suppression of TGF-β1/Smad signaling, reduced EMT/ECM accumulation, and enhanced Nrf2/HO-1 activation (Table 1). These findings point to a therapeutic window in which efficacy can be improved without compromising safety. Considering between-study differences in model background, dosing route and duration, and formulation, future preclinical studies should implement standardized dosing frameworks and uniform efficacy/safety endpoints to more precisely define exposure–response relationships and guide clinical dose selection.

## 4 Current clinical application status and challenges

Antioxidants can mitigate oxidative stress by either inhibiting enzymes responsible for ROS production or chelating trace elements involved in free radical generation, thereby reducing the risk of oxidative damage and disease development. Quercetin, a naturally occurring flavonoid antioxidant, effectively scavenges free radicals, modulates immune responses through multiple signaling pathways, regulates enzymatic activity, alters cell cycle progression, and influences gene expression. Through these mechanisms, Quercetin exerts protective effects at all stages of pathogenesis by inhibiting, reversing, or delaying disease progression.

Quercetin exhibits a broad spectrum of biological activities and regulates numerous intra- and extracellular signaling pathways implicated in disease progression. Despite its pharmacological advantages, its instability in physiological media, short biological half-life, and extremely low oral bioavailability remain major barriers to clinical translation (Almeida et al., 2018). Increasing evidence indicates that synthesizing novel Quercetin derivatives can improve its poor solubility and bioavailability. Structural modifications—such as hydroxyl etherification or esterification, carbonyl substitution, and functionalization of the A- and B-rings—have yielded derivatives with enhanced solubility, stability, and bioavailability, as well as improved antioxidant, anti-inflammatory, and anticancer activities (Massi et al., 2017; Sharma et al., 2018; Rajaram et al., 2019).

Given these challenges, recent research has shifted toward multidimensional optimization strategies that combine chemical modification, metal complex formation, and advanced drug delivery systems to overcome Quercetin’s pharmacokinetic limitations and maximize its therapeutic potential, particularly in the treatment of DN.

### 4.1 Structural modification and delivery strategies

Quercetin, as a multitarget and multipathway natural antioxidant, has shown promising pharmacological activity in various chronic diseases, especially DN. However, its instability in physiological environments, short half-life, and poor oral bioavailability significantly limit its clinical utility (Almeida et al., 2018). To address these issues, three main approaches have been investigated: (1) chemical structural modification, (2) metal complex formation, and (3) advanced drug delivery systems.

#### 4.1.1 Chemical structural modification

Chemical modifications targeting hydroxyl and carbonyl groups, as well as the A- and B-ring structures of Quercetin, have been employed to improve its solubility, stability, and biodistribution. Typical strategies include hydroxyl etherification and esterification, carbonyl substitution, and aromatic ring functionalization. These modifications have not only significantly enhanced water solubility and oral bioavailability but also, in some derivatives, augmented antioxidant, anti-inflammatory, and antitumor activities (Massi et al., 2017; Sharma et al., 2018; Rajaram et al., 2019). Such molecular tailoring provides a rational foundation for optimizing Quercetin’s pharmacodynamics in chronic disease therapy.

#### 4.1.2 Metal complex strategy

Metal ions and their complexes have broad applications in drug development and clinical practice due to their capacity to improve stability, increase target specificity, and modulate redox homeostasis. Refat et al. (2021) investigated the antidiabetic activity of a quercetin–zinc(II) complex (13a) in a STZ-induced diabetic rat model. Their findings revealed that combination therapy with 13a and mesenchymal stem cells (MSCs) markedly enhanced insulin secretion, suppressed inflammatory responses, improved pancreatic injury and glucose metabolism abnormalities, and exhibited superior efficacy compared with either treatment alone. Additionally, this regimen effectively reduced hyperglycemia and genotoxicity, highlighting the potential of Quercetin–metal complexes for managing diabetes and its complications.

#### 4.1.3 Advanced drug delivery systems

Nanotechnology offers an innovative solution to overcome Quercetin’s poor oral bioavailability. An ideal delivery system should protect Quercetin from degradation in the upper gastrointestinal tract, achieve targeted release in the colon, provide sustained release, and significantly improve *in vivo* stability (Liu et al., 2025). Nanoparticles—owing to their high surface area, tunable targeting capabilities, protection against enzymatic degradation, and single-dose controlled-release properties—have emerged as a highly promising platform for Quercetin delivery (Sarkar et al., 2002; Rizvi and Saleh, 2018). Barbosa et al. (2019) developed polymeric Quercetin nanoparticles via polyelectrolyte self-assembly using fucoidan and chitosan as carriers. This nanosystem demonstrated excellent antioxidant activity and sustained-release characteristics in simulated gastrointestinal environments, particularly under 3F/1C and 5F/1C conditions, showing remarkable tolerance to pH fluctuations and effectively preventing Quercetin degradation. Nonetheless, research on Quercetin nanodelivery specifically for DN remains limited, and further comprehensive studies on pharmacokinetics, biodistribution, and mechanisms of action are warranted.

In conclusion, chemical modification, metal complex formation, and advanced drug delivery technologies provide complementary strategies to optimize Quercetin for clinical application in DN. Future efforts should integrate mechanistic insights with targeted delivery and intelligent release platforms to accelerate the translation of Quercetin from laboratory research to clinical practice.

## 5 Discussion and perspectives

DN is a multifactorial and complex disease involving diverse pathological processes, including metabolic disorders, oxidative stress, inflammatory responses, apoptosis, and fibrosis. Current clinical treatments can delay disease progression to some extent but remain insufficient to reverse established structural and functional damage to the kidneys. Therefore, identifying therapeutic agents with broad targets, low toxicity, and suitability for long-term intervention remains a critical priority in the prevention and treatment of DN.

**FIGURE 4:**
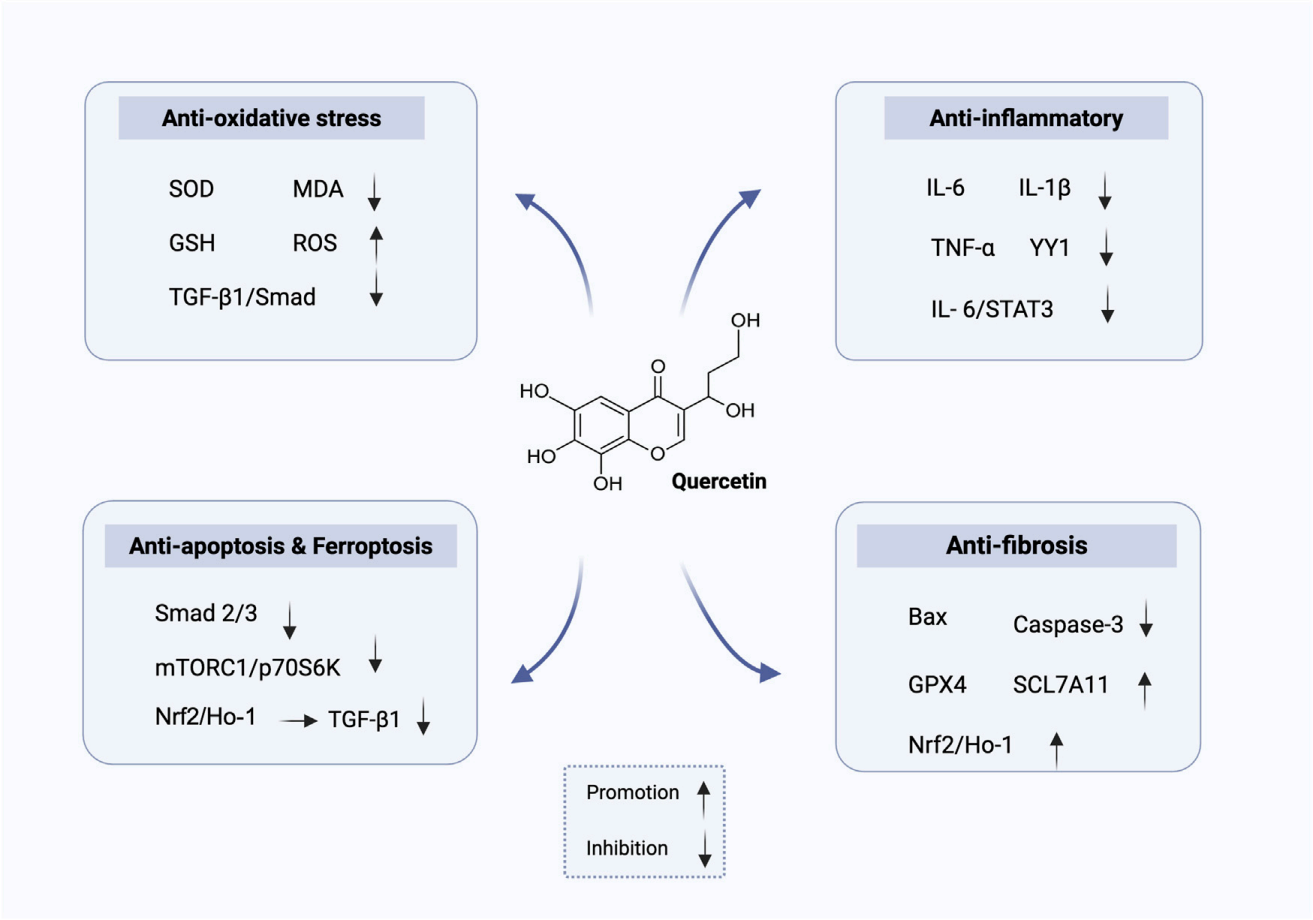
Mechanisms of quercetin in the treatment of DN.

Quercetin, a naturally occurring flavonoid, exhibits multi-target regulatory properties and a favorable safety profile, demonstrating considerable potential in multi-pathway intervention for DN. As shown in Figure 4, quercetin exerts renoprotective effects through anti-oxidative stress, anti-inflammatory, anti-fibrotic, and anti-apoptotic/ferroptotic mechanisms. A growing body of experimental evidence indicates that quercetin not only mitigates renal injury through its antioxidant and anti-inflammatory activities but also suppresses TGF-β1–mediated fibrosis, preserves podocyte function, and, under certain conditions, induces ferroptosis, thereby impeding disease progression. Notably, these mechanisms may act in a synergistic manner, suggesting that quercetin possesses an integrative capacity to modulate the disease network in DN.

Despite abundant preclinical evidence supporting its therapeutic potential, the clinical translation of quercetin remains challenging. First, its low oral bioavailability and limited absorption hinder the maintenance of effective concentrations in target tissues. Second, variations in efficacy across different experimental models and dosing regimens highlight the need to determine optimal therapeutic doses and administration strategies. Third, there is a lack of high-quality, long-term randomized controlled trials to confirm its safety and efficacy. Future research should focus on: (i) enhancing the solubility and tissue targeting of quercetin through strategies such as nanoparticle-based delivery systems, structural modification, and co-delivery with other agents; (ii) applying multi-omics technologies and network pharmacology to elucidate its multi-target, multi-pathway mechanisms and the interplay between them; (iii) conducting multicenter, large-sample clinical trials to assess its efficacy and safety across different stages of DN, both as monotherapy and in combination with conventional treatments; and (iv) exploring synergistic effects between quercetin and other natural compounds or existing drugs to develop more effective combination therapies.

In summary, quercetin holds broad application prospects in DN therapy. Its multi-target, multi-pathway regulatory features provide novel insights for the integrated management of complex diseases. With continued advancements in drug delivery technologies and precision medicine, quercetin is expected to achieve successful translation from bench to bedside, offering safer and more effective therapeutic options for patients with DN.
